# Research landscape of photodynamic therapy for hepatocellular carcinoma: Hotspots and Prospects from 2012 to 2025

**DOI:** 10.1186/s41065-025-00509-1

**Published:** 2025-08-26

**Authors:** Fei Yu, Sihan Yin, Jiayu Zhu, Kewei Sun

**Affiliations:** Department of Hepatology of the First Affiliated Hospital, Hunan University of Chinese Medicine, Hunan University of Chinese Medicine, Yuelu District, 300 Xueshi Road, Science & Education Park, Changsha, 410205 Hunan China

**Keywords:** Bibliometric analysis, Hepatocellular carcinoma, Photodynamic therapy, Nanotechnology, Photosensitizer, Combination therapy

## Abstract

**Objective:**

To explore the current status and future development of photodynamic therapy (PDT) for hepatocellular carcinoma (HCC). However, this field lacks a comprehensive bibliometric analysis. This study aims to investigate the research content and hotspots in PDT for liver cancer from 2012 to 2025, and to predict future research directions, providing references for subsequent studies.

**Methods:**

We chose the Web of Science Core Collection (WoSCC) database and retrieved articles published in the field between 2012 and 2025. Bibliometric and visualization analyses were performed using R (version 4.4.1), VOSviewer (version 1.6.19), and CiteSpace (version 6.4. R1).

**Results:**

A total of 547 papers were included. We found that the number of publications in this field has steadily increased from 2012 to 2025. China leads with the highest number of publications, followed by the USA, Korea, Germany, and Japan. China has the lowest international co-authorship rate, while Germany and Japan show higher international collaboration rates. The *International Journal of Nanomedicine* is the most popular journal for publication, whereas *Biomaterials* ranks first in terms of citations. Our analysis of keywords and the most cited references revealed that current research focuses on the mechanism of PDT-induced apoptosis in HCC, the development of photosensitizers (PSs), nanotechnology-enhanced PDT, and synergistic treatment of HCC with PDT and other therapies. Nanotechnology and multimodal synergistic therapeutic strategies are driving the treatment of HCC.

**Conclusion:**

PDT, as a therapy for HCC, is expected to become a research hotspot. This paper analyzes PDT's current research for HCC, offering references for future research in related fields.

**Supplementary Information:**

The online version contains supplementary material available at 10.1186/s41065-025-00509-1.

## Introduction

Primary liver cancer comprises three distinct pathologies: Hepatocellular carcinoma (HCC), Intrahepatic cholangiocarcinoma (ICC), and Combined hepatocellular-cholangiocarcinoma (cHCC-CCA). HCC accounts for about 90% of all primary liver cancer cases [1]. According to Global Cancer Statistics 2020, primary liver cancer is the sixth most commonly diagnosed cancer and the third leading cause of cancer death worldwide [2]. Projections suggest that the incidence of HCC will rise significantly, with a 137% increase in morbidity and a 178% increase in mortality by 2030  [3].The incidence of HCC has stabilized in China, while in many countries across Europe, North America, and South America, the incidence continues to rise, possibly linked to METS  [4, 5].

Surgery remains the first-line treatment for most malignant tumors, especially for primary liver cancer. After surgical resection of the tumor, adjuvant therapies such as chemotherapy, targeted therapy, and immunotherapy are also critical. These treatments, however, come with limitations, including side effects, poor pharmacokinetics, and inadequate biodistribution. As a result, the traditional approach of direct drug administration is becoming less clinically applicable [6]. Photodynamic therapy (PDT) has shown promise in enhancing the detection rate of microscopic lesions through fluorescence-targeted visualization, which has proven to be both safe and feasible [7]. It works by selectively enriching PS in tumor tissues and generating reactive oxygen species (ROS) when exposed to specific wavelengths of light, thereby killing cancer cells. Because PDT causes minimal damage to surrounding healthy liver tissue, it is primarily applied to patients with early-stage, localized HCC, especially those who are unable to tolerate conventional treatments such as surgery, ablation, or other contraindicated therapies. Furthermore, PDT has the potential to activate an anti-tumor immune response, which could reduce the risk of recurrence. PDT can also be combined with other therapies to optimize treatment regimens for HCC. Therefore, exploring the clinical application of PDT, especially in combination with existing treatments, is crucial for improving patient outcomes.

Currently, neither the U.S. NCCN Guideline (2025) (https://www.nccn.org/professionals/physician gls/pdf/hcc.pdf) nor the European EASL Guideline (2024) [8]. includes PDT in the standard diagnostic and therapeutic recommendations for HCC, primarily because most supporting evidence is derived from small-sample studies. The Japanese JSH Guideline (2021) [9]. permits the cautious use of PDT as a palliative treatment in select cases, such as patients with Child–Pugh class C liver function or tumors located adjacent to major blood vessels, following evaluation by a multidisciplinary team. Although China’s Primary Liver Cancer Guidelines (2024 edition) [10]. do not list PDT as a standard treatment, they mention its use under “special case management.” Specifically, PDT is considered suitable for patients with severe cirrhosis or tumors adjacent to blood vessels or bile ducts who cannot tolerate invasive treatments. This discrepancy highlights the need for research on PDT for the treatment of HCC, particularly to facilitate a consensus on international clinical guidelines.

Bibliometrics, as a scientific quantitative analysis tool, reveals development trends, hot topics, and frontiers in specific research fields through statistical analysis of scientific literature [11]. This study, therefore, employs bibliometric methods to comprehensively analyze research on the application of photodynamic techniques in HCC. The aim is to provide valuable insights for scholars and clinicians in this field and promote further development.

## Materials and methods

### Data collection

The data for this study were obtained from the Web of Science Core Collection (WoSCC; Hunan University of Chinese Medicine licensed edition) on April 25, 2025. WoSCC was selected for two primary reasons: first, it offers a high-quality and comprehensive range of indexed journals; and second, it is widely recognized and frequently utilized in bibliometric research, providing a robust and credible foundation for analysis. To ensure accuracy and consistency, two researchers independently conducted the search on the same day and during the same period. The final search strategy was as follows: TS = (photochemo* OR"Photodynamic action"OR"Photodynamic therapy*"OR Photosensitizer) AND TS = ("liver cancer*"OR"liver tumor*"OR"Liver Carcinoma*"OR"Liver Neoplasm*"OR"Hepatic Neoplasm*"OR"Hepatic cancer*"OR"Hepatic tumor*"OR"Hepatocellular Cancer*"OR"Hepatocellular Carcinoma*"OR"Hepatoma*"OR"liver and intrahepatic bile duct carcinoma"OR"liver and intrahepatic biliary tract cancer"OR"Intrahepatic holangiocarcinoma"OR"Combined epatocellular-cholangiocarcinoma") AND DOP = (2012–01-01/2025–04-25) AND DT = (Article OR Review) AND LA = (English). After excluding irrelevant and duplicate records, a total of 547 relevant articles were identified. These records were exported in plain text format with full citation information, including cited references. Notably, the terms “liver and intrahepatic bile duct carcinoma,” “liver and intrahepatic biliary tract cancer,” “Intrahepatic holangiocarcinoma,” and “Combined epatocellular-cholangiocarcinoma” all returned identical results in terms of the number of documents retrieved. Therefore, for clarity and consistency, the term “liver cancer” in this study is uniformly referred to as HCC.

## Data analysis

We adopted established methodologies from previous bibliometric research studies [12]. In this study, OriginPro 2025 software was used to analyze annual publication trends. Additionally, R software (version 4.4.1) with the bibliometrix package (version 4.0), VOSviewer (version 1.6.19), and CiteSpace (version 6.4. R1) were employed for bibliometric data analysis and visualization. Journal impact factor (IF) data were obtained from the 2023 edition of the Journal Citation Reports (JCR). VOSviewer was used to generate a range of visualizations, including co-authorship networks by country and institution, co-citation networks, and keyword co-occurrence maps. In constructing the co-authorship network, we included only countries with at least two publications and institutions with at least three. For co-citation analysis, we considered documents cited 35 times or more. The keyword co-occurrence analysis included only those keywords appearing in at least five publications, while excluding generic terms such as “photodynamic,” “liver cancer,” and their synonyms. CiteSpace was primarily employed to uncover the knowledge structure, research hotspots, and evolutionary trends within the field. Accordingly, we used these tools to systematically analyze the research landscape of PDT in HCC and to forecast future research directions.

## Results

### General landscapes of included documents on PDT and HCC

**Fig. 1:**
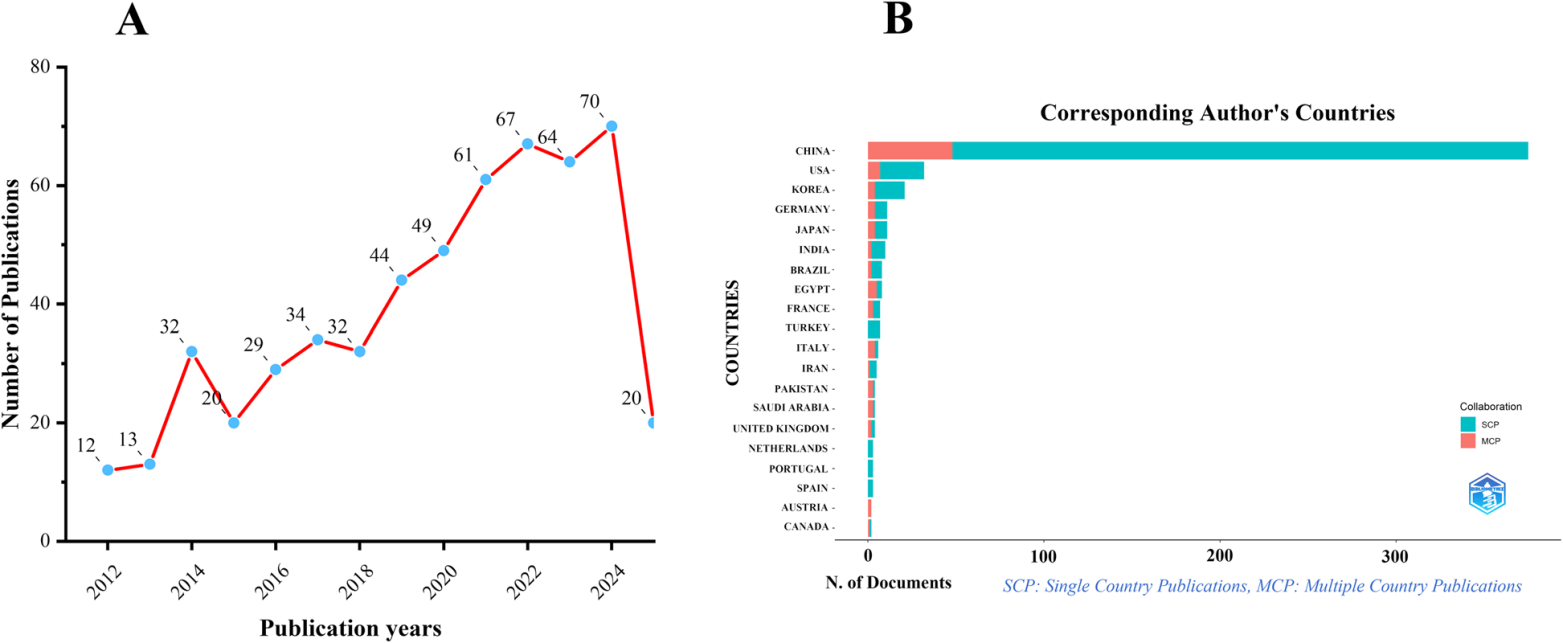
Annual publication output trends in PDT for HCC from 2012 to 2025. **A** Trends of annual publication outputs. **B** Distribution of corresponding authors’ countries and cooperation

After searching the WoSCC database, we identified a total of 547 non-duplicate publications related to PDT and HCC. As shown in Fig. 1A, the number of publications steadily increased from 2012 to 2024. For 2025, data were available only through April, with 20 publications recorded thus far.

Based on the affiliation of the corresponding author, we found that China produced the highest number of publications (*n* = 375), followed by the United States (*n* = 32), South Korea (*n* = 21), Germany (*n* = 11), and Japan (*n* = 11). This distribution may be attributed to the relatively high incidence of HCC in China, along with advancements in nanophotosensitizers and targeted delivery technologies. It is also noteworthy that China exhibits the lowest rate of international co-authorship (12.8%) among the top five publishing countries, whereas Germany and Japan report the highest rates (36.4%). The USA and South Korea follow with co-authorship rates of 21.9% and 19.0%, respectively. These findings suggest that China would benefit from expanding its international research collaborations to promote further development in this field, as illustrated in Fig. 1B and Table 1.

**Table 1:** Most relevant countries by corresponding authors in PDT for HCC

Country	Articles	Articles %	SCP	MCP	MCP %
CHINA	375	68.6	327	48	12.8
USA	32	5.9	25	7	21.9
KOREA	21	3.8	17	4	19
GERMANY	11	2	7	4	36.4
JAPAN	11	2	7	4	36.4
INDIA	10	1.8	8	2	20
BRAZIL	8	1.5	6	2	25
EGYPT	8	1.5	3	5	62.5
FRANCE	7	1.3	4	3	42.9
TURKEY	7	1.3	7	0	0
ITALY	6	1.1	2	4	66.7
IRAN	5	0.9	4	1	20
PAKISTAN	4	0.7	1	3	75
SAUDI ARABIA	4	0.7	1	3	75
UNITED KINGDOM	4	0.7	2	2	50
NETHERLANDS	3	0.5	3	0	0
PORTUGAL	3	0.5	3	0	0
SPAIN	3	0.5	3	0	0
AUSTRIA	2	0.4	0	2	100
CANADA	2	0.4	1	1	50

*MCP* multiple-country publication, *SCP* single-country publication

**Fig. 2:**
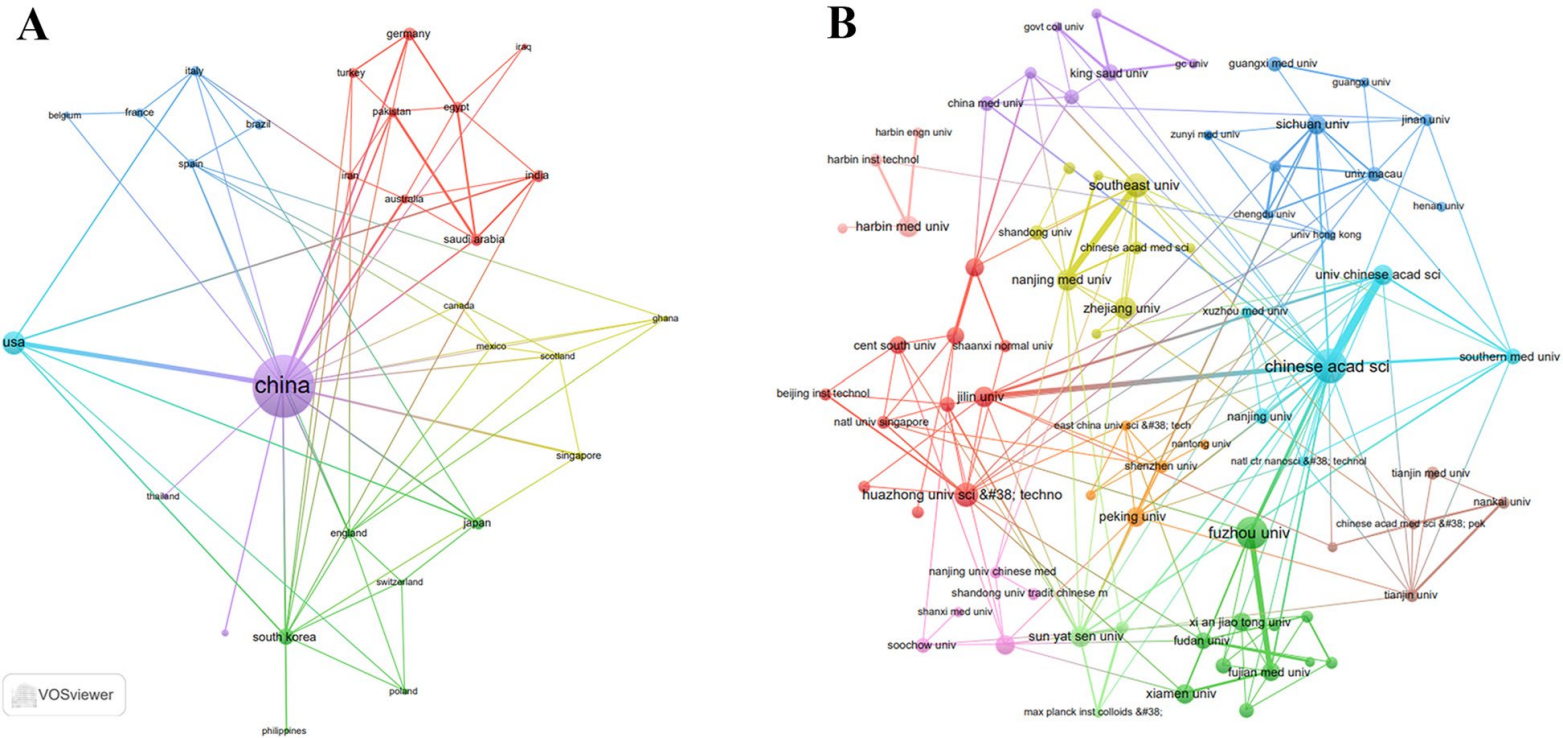
Map of cooperation between different institutions.Collaboration network of countries/regions and institutions in PDT for HCC from 2012 to 2025. **A** Map of cooperation between different countries. **B** Map of cooperation between different institutions

In addition, Fig. 2A shows that China maintains the most extensive international collaborations in the field of PDT for HCC. The collaboration network highlights the Chinese Academy of Sciences (*n* = 35) and Fuzhou University (*n* = 31) as leading institutions with significant research output and collaborative activity (Fig. 2B; Table 2).

**Table 2:** Most relevant affiliations of PDT for HCC

Affiliation	Articles (n)
Chinese Academy of Sciences	35
Fuzhou University	31
Huazhong University of Science and Technology	17
Southeast University	17
zhejiang University	15
Harbin Medical University	14
Nanjing Medical University	13
Sun Yat-sen University	13
Jilin University	12
University of Chinese Academy of Sciences	12
Peking University	11
Shanghai Jiao Tong University	11
Sichuan University	11
Xiamen University	11
The Chinese University of Hong Kong	10
Fujian Medical University	10
Xian Jiao Tong University	10
Central South University	9
City University of Hong Kong	9
Fudan University	8

## Journal analysis and visualization

Using R software (version 4.4.1) with the bibliometrix and ggplot2 packages, we analyzed the journals with the highest number of publications and the most frequently cited journals in the field of PDT for HCC. Additionally, VOSviewer (version 1.6.19) was employed for co-citation journal analysis. The results revealed that the 547 articles included in this study were distributed across 268 different academic journals (Annex 1).

**Fig. 3:**
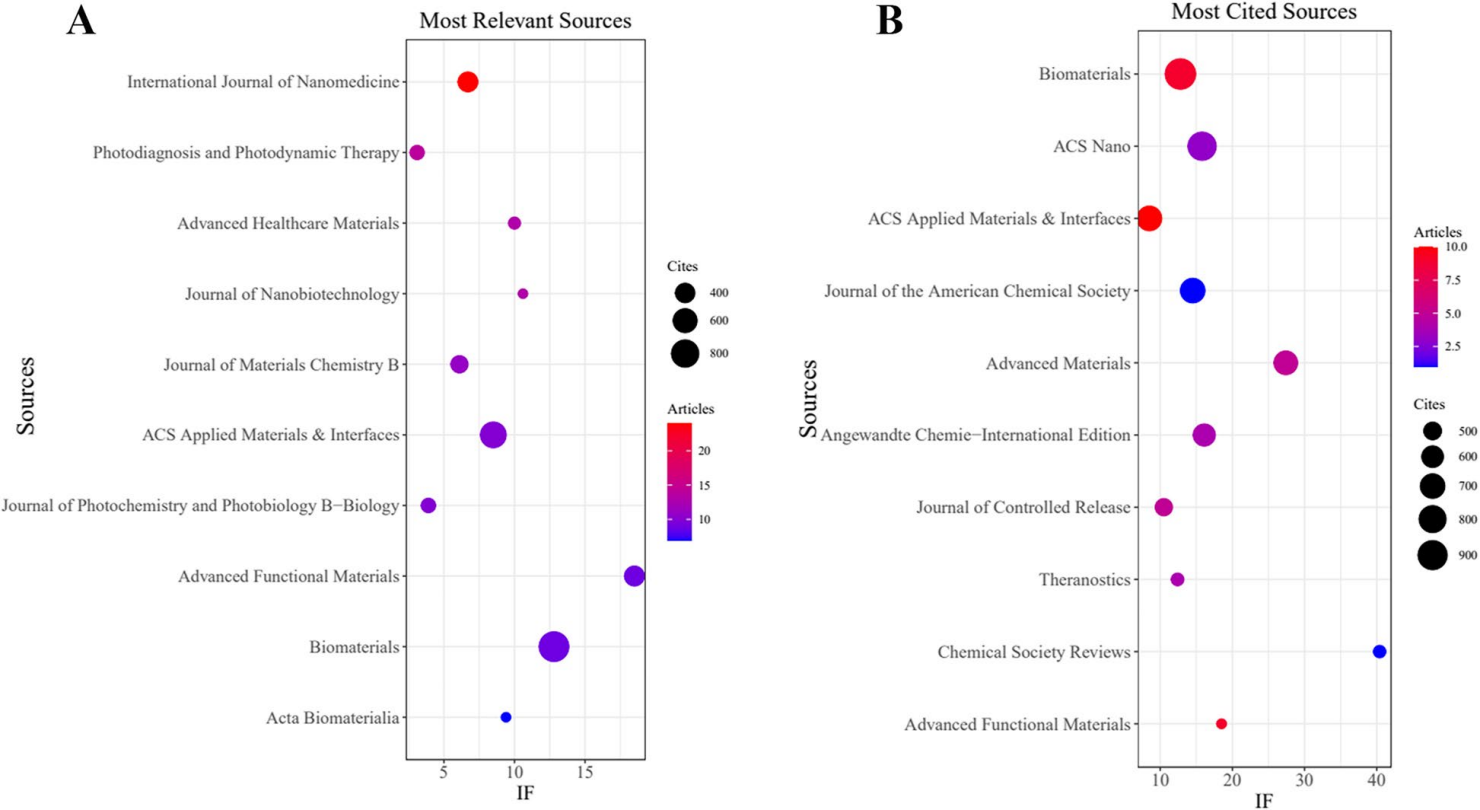
Journal with the largest number of articles published and the journal with the largest number of citations. **A** Journal with the largest number of articles published. **B** Journals with the largest number of citations

Regarding publication frequency, the most prominent journals are listed in Table 3 and Fig. 3A. The most popular journals are International Journal of Nanomedicine (*n* = 24, IF = 6.7), followed by Photodiagnosis and Photodynamic Therapy (*n* = 14, IF = 3.1), Advanced Healthcare Materials (*n* = 13, IF = 10.0), Journal of Nanobiotechnology (*n* = 13, IF = 10.6), and Journal of Materials Chemistry B (*n* = 11, IF = 6.1).

**Table 3:** Top 10 journals with the most published articles

Journal	Documents	IF (2023)	Cites
International Journal of Nanomedicine	24	6.7	415
Photodiagnosis and Photodynamic Therapy	14	3.1	262
Advanced Healthcare Materials	13	10	231
Journal of Nanobiotechnology	13	10.6	219
Journal of Materials Chemistry B	11	6.1	329
ACS Applied Materials & Interfaces	10	8.5	705
Journal of Photochemistry and Photobiology B-Biology	10	3.9	264
Advanced Functional Materials	9	18.5	419
Biomaterials	9	12.8	971
Acta Biomaterialia	7	9.4	219

In addition to identifying journals with the highest publication output, we also analyzed the most frequently cited journals, as shown in Table 4 and Fig. 3B. Biomaterials was the most cited journal (*n* = 971 citations, IF = 12.8), followed by ACS Nano (*n* = 881, IF = 15.8), ACS Applied Materials & Interfaces (*n* = 705, IF = 8.5), Journal of the American Chemical Society (*n* = 702, IF = 14.5), and Advanced Materials (*n* = 668, IF = 27.4). A comparison of publication volume and citation frequency indicates that the journals with the most published articles are not necessarily the most highly cited. This discrepancy may be attributable to differences in journal impact, article types, or subject focus.

**Table 4:** Top 10 journals with the most cited journals

Journal	Cites	IF (2023)	Documents
Biomaterials	971	12.8	9
ACS Nano	861	15.8	3
ACS Applied Materials & Interfaces	705	8.5	10
Journal of the American Chemical Society	702	14.5	1
Advanced Materials	668	27.4	5
Angewandte Chemie-International Edition	616	16.1	4
Journal of Controlled Release	492	10.5	5
Theranostics	430	12.4	4
Chemical Society Reviews	428	40.4	1
Advanced Functional Materials	419	18.5	9

**Fig. 4:**
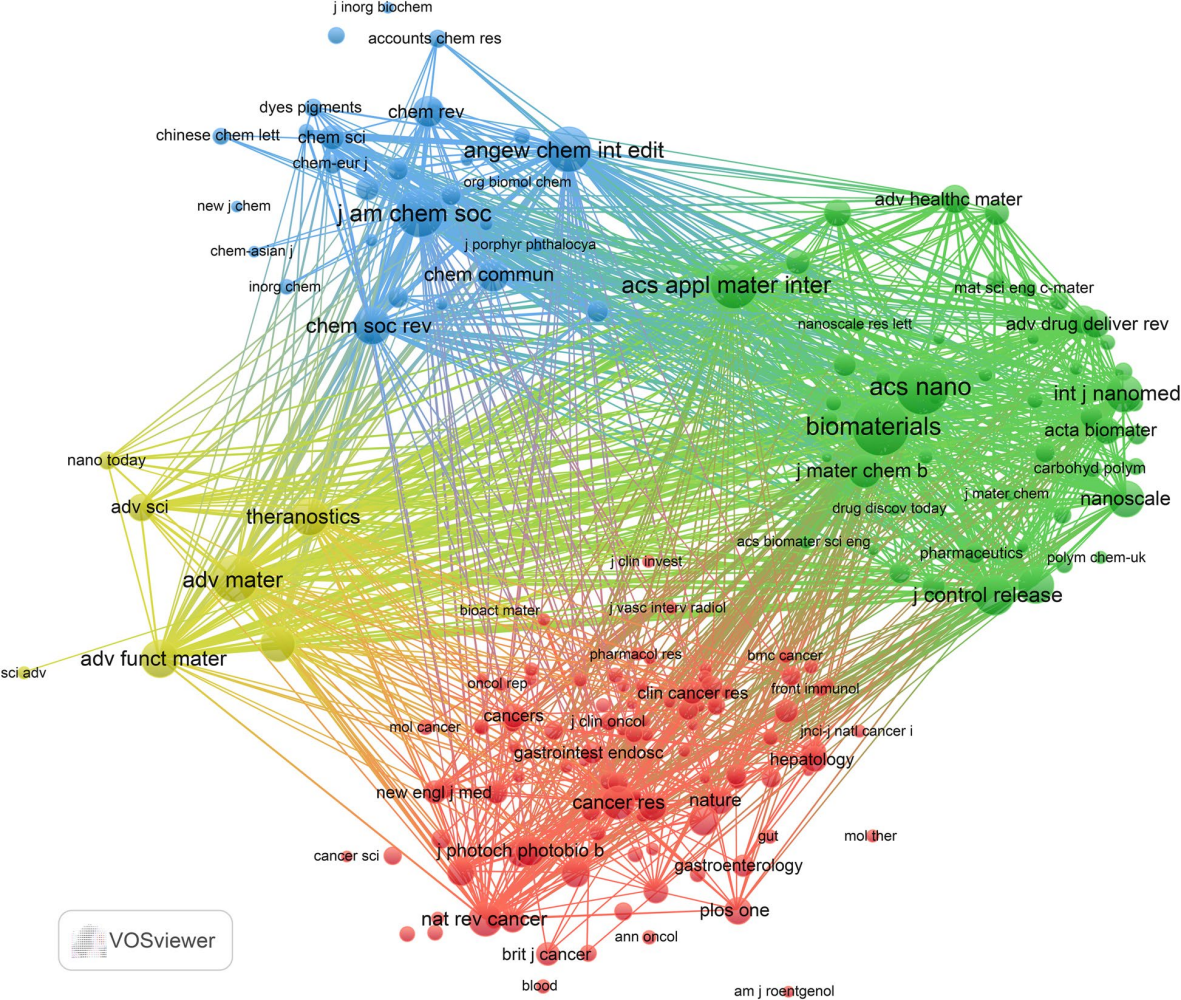
Co-cited journals involved in PDT for HCC

The journal co-citation analysis, visualized in Fig. 4, highlights Biomaterials, ACS Nano, and ACS Applied Materials & Interfaces as particularly influential within the PDT-HCC research landscape. However, these findings also suggest a relative scarcity of high-impact publications reporting clinical or translational outcomes of PDT in HCC. This underscores the need for more in-depth and high-quality research in this field to elevate its academic visibility and influence.

## Most cited references and reference burst

Using the bibliometrix package in R software, we identified the 20 most-cited articles in the field of PDT for HCC, each of which has been cited more than 140 times and collectively spans 15 different academic journals (Table 5). Based on citation volume and journal distribution, it appears that no single theoretical breakthrough has yet defined the field. The top three most-cited articles were: “Silver nanoparticles: synthesis, properties, and therapeutic applications,” “H₂S-activatable near-infrared afterglow luminescent probes for sensitive molecular imaging in vivo,” and “Emerging strategies of nanomaterial-mediated tumor radiosensitization.”A thematic analysis of these 20 papers reveals that they can be broadly categorized into four main research areas:(1) Development of novel PS;(2) Application of nanomaterials to enhance PDT efficacy;(3) Combination therapies incorporating PDT to suppress HCC progression; (4) Targeted delivery systems aimed at enabling precision medicine.

**Table 5:** Top 20 cited references related to PDT for HCC

Paper	DOI	Total Citations	TC per Year
WEI LY, 2015, DRUG DISCOV TODAY	10.1016/j.drudis.2014.11.014	685	62.27
WU LY, 2020, NAT COMMUN	10.1038/s41467-020–14307-y	562	93.67
XIE JN, 2019, ADV MATER	10.1002/adma.201802244	312	44.57
HU F, 2014, ANAL CHEM	10.1021/ac502103t	259	21.58
BONNET S, 2018, DALTON T	10.1039/c8dt01585f	222	27.75
SHI CH, 2016, J BIOMED OPT	10.1117/1.JBO.21.5.050901	217	21.70
XIA L, 2014, BIOMATERIALS	10.1016/j.biomaterials.2014.01.068	203	16.92
BRODOWSKA K, 2014, EXP EYE RES	10.1016/j.exer.2014.04.011	193	16.08
HUANG L, 2021, BIOMATERIALS	10.1016/j.biomaterials.2020.120557	192	38.40
LI XS, 2018, CHEM SCI	10.1039/c7sc05115h	183	22.88
WANG SW, 2019, ACS NANO	10.1021/acsnano.8b08398	182	26.00
ZHANG MH, 2023, SIGNAL TRANSDUCT TAR	10.1038/s41392-023–01382-y	180	60.00
WANG Y, 2017, ADV MATER	10.1002/adma.201605357	176	19.56
YANG YY, 2021, BIOMATERIALS	10.1016/j.biomaterials.2020.120456	174	34.80
LUO GF, 2016, ADV FUNCT MATER	10.1002/adfm.201505175	158	15.80
SHIRATA C, 2017, SCI REP-UK	10.1038/s41598-017–14401-0	155	17.22
DU JB, 2021, THERANOSTICS	10.7150/thno.59121	154	30.80
LI ZL, 2018, ADV FUNCT MATER	10.1002/adfm.201800145	145	18.13
SUN TT, 2019, ACS NANO	10.1021/acsnano.9b03910	143	20.43
LI Y, 2017, ADV HEALTHC MATER	10.1002/adhm.201600924	143	15.89

**Fig. 5:**
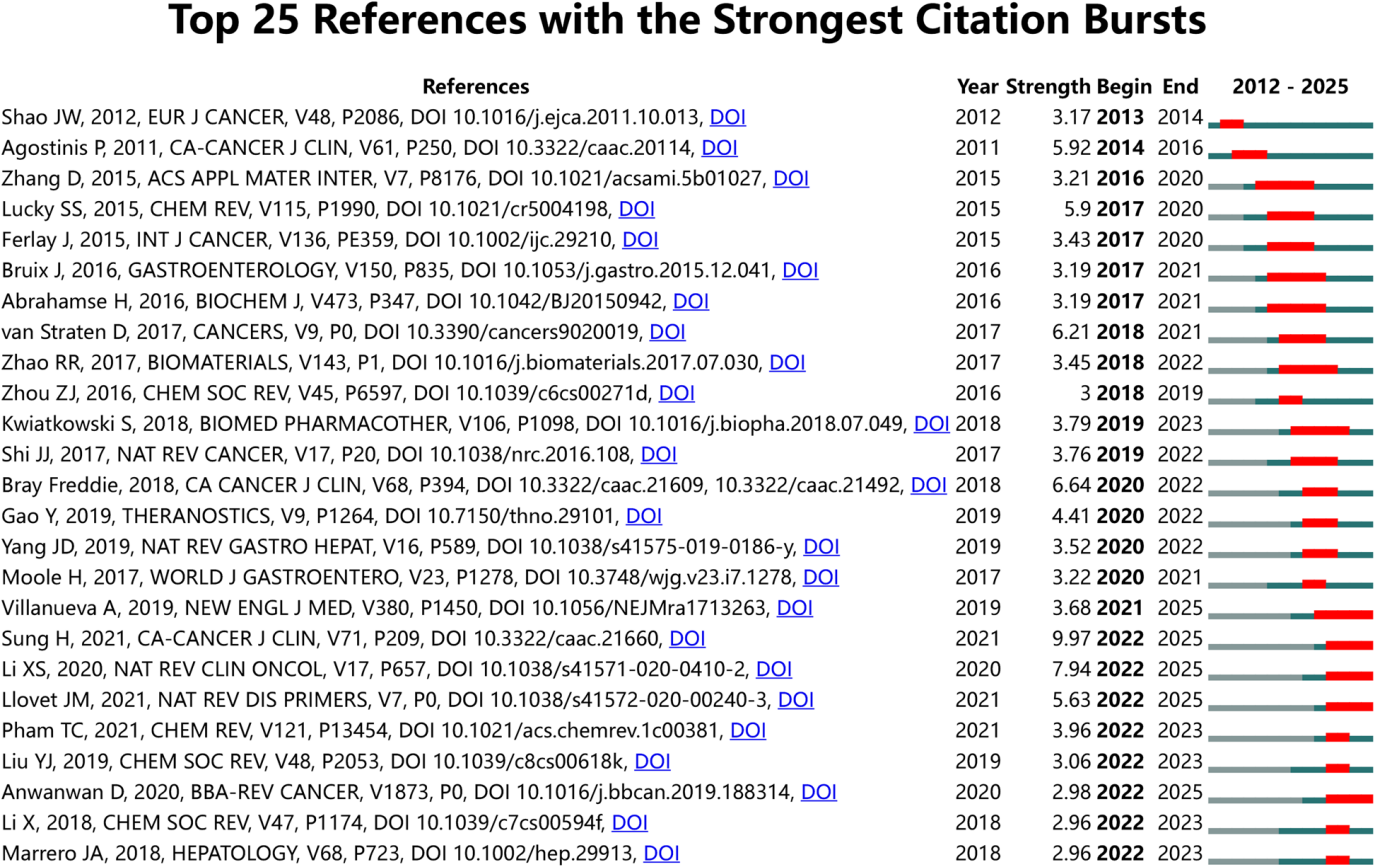
Top 25 references with the strongest citation bursts in PDT for HCC

To further examine citation dynamics, we used CiteSpace to identify the top 25 references exhibiting significant citation bursts. The detection criteria included: top 25 citations by burst strength, a minimum burst duration of two years, and a status count of two. The resulting references are visualized in Fig. 5, with full titles and DOIs listed in Annex 2. The citation burst intensities for these articles ranged from 2.96 to 9.97. Notably, the strongest bursts were observed in the following works: “Global Cancer Statistics 2020: GLOBOCAN Estimates of Incidence and Mortality Worldwide for 36 Cancers in 185 Countries” (strength: 9.97); “Clinical Development and Potential of Photothermal and Photodynamic Therapies for Cancer” (strength: 7.94); “Global Cancer Statistics 2018: GLOBOCAN Estimates of Incidence and Mortality Worldwide for 36 Cancers in 185 Countries” (strength: 6.64). It is noteworthy that the three leading citation bursts are “Diagnosis, Staging, and Management of Hepatocellular Carcinoma: 2018 Practice Guidance by the American Association for the Study of Liver Diseases”, “Supramolecular photosensitizers rejuvenate photodynamic therapy”, and “Challenges in liver cancer and possible treatment approaches”.

The results indicate that the majority of the literature focuses on nanomaterial-enhanced PS and the integration of PDT with other modalities, such as chemotherapy or immunotherapy, for the multimodal treatment of HCC.

## Keyword clusters and evolution of themes

Keyword clustering provides a rapid means of identifying the primary themes and research directions within a given field. In this study, a total of 2,591 keywords were extracted using VOSviewer. Table 6 presents the top 20 most frequently occurring keywords, each appearing more than 20 times. The most frequent keyword was “nanoparticles” (*n* = 130), followed by “apoptosis” (*n* = 77), “co-delivery” (*n* = 74), “in vitro” (*n* = 63), “drug delivery” (*n* = 59), and “cell” (*n* = 58).

**Table 6:** The top 20 keywords

Rank	Keywords	Count
1	nanoparticles	130
2	apoptosis	77
3	co-delivery	74
4	in-vitro	63
5	drug delivery	59
6	cell	58
7	therapy	42
8	chemotherapy	41
9	combination therapy	38
10	reactive oxygen species	35
11	cancer therapy	34
12	in-vivo	34
13	cancer cells	32
14	mechanism	29
15	doxorubicin	27
16	sorafenib	26
17	diagnosis	24
18	indocyanine green	24
19	immunotherapy	23
20	radiofrequency ablation	21

**Fig. 6:**
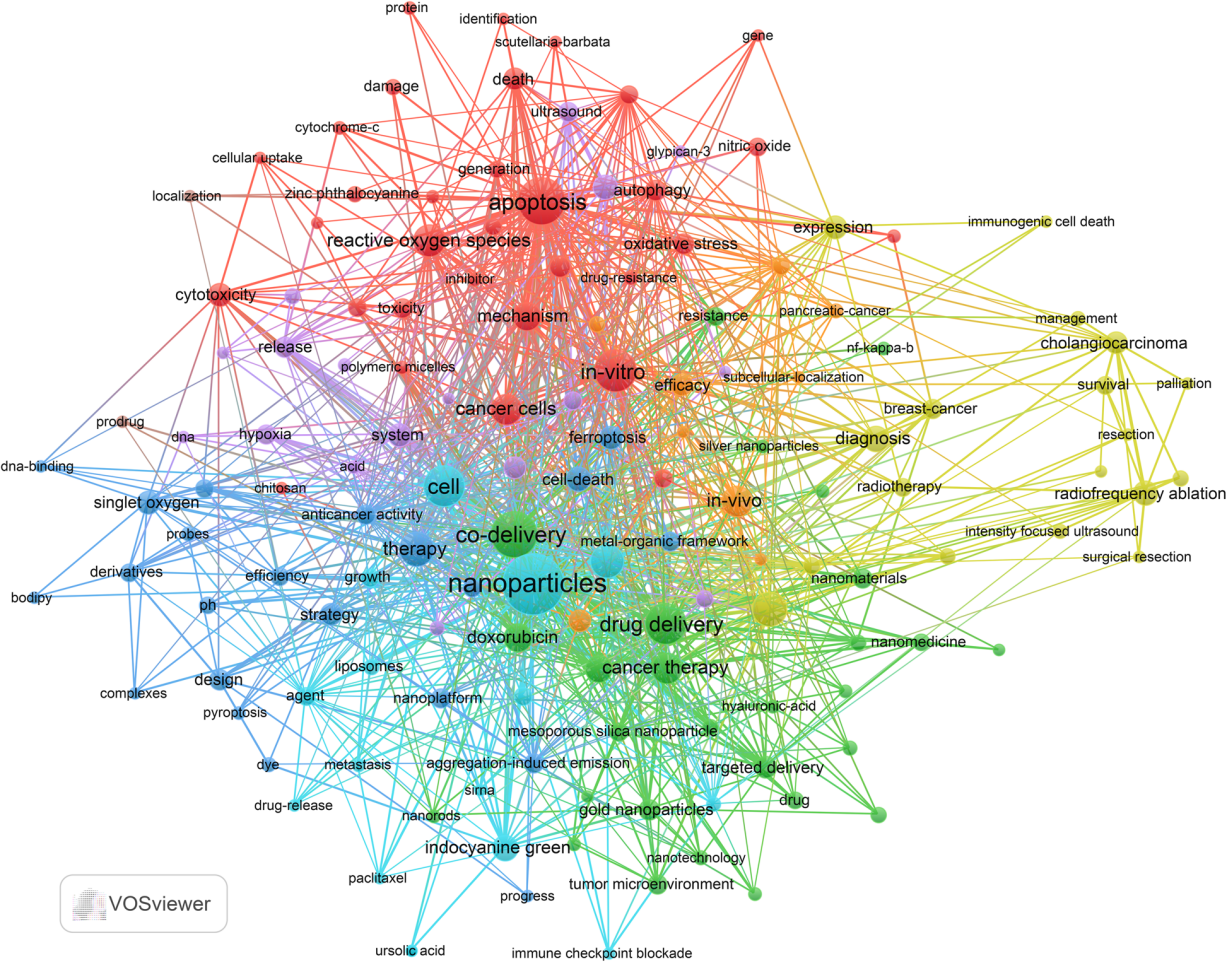
Keyword co-occurrence map of publications on the relationship between PDT and HCC

Based on these data, we selected 193 keywords that appeared at least five times to generate a keyword cluster map (Fig. 6). We then identified and analyzed six major clusters, each containing no fewer than 15 keywords: Mechanisms of PDT-induced apoptosis (Red Cluster) – This cluster contains 41 keywords, including apoptosis, in vitro, reactive oxygen species, in vivo, and cancer cells. Nanoparticle and carrier systems for delivery of PSs or drugs to HCCs (Green Cluster) – Includes 28 keywords such as co-delivery, drug delivery, cancer therapy, doxorubicin, and sorafenib. Chemical design of PSs (Blue Cluster) – Comprises 22 keywords including therapy, cell death, ferroptosis, singlet oxygen, and strategy. Sonodynamic–Photodynamic Synergy and Hypoxia Microenvironment Intervention (Yellow Cluster) – Contains 19 keywords such as sonodynamic therapy, system, upconversion nanoparticles, release, and hypoxia. Integration of PDT with Conventional Therapies (Purple Cluster) – Consists of 18 keywords, including chemotherapy, diagnosis, radiofrequency ablation, expression, and cholangiocarcinoma. Nano-Immunotherapy Combinations and Antimetastatic Strategies (Cyan Cluster) – Comprises 15 keywords, including nanoparticles, cell, combination therapy, indocyanine green, and agent (see Annex 3 for the full list).

**Fig. 7:**
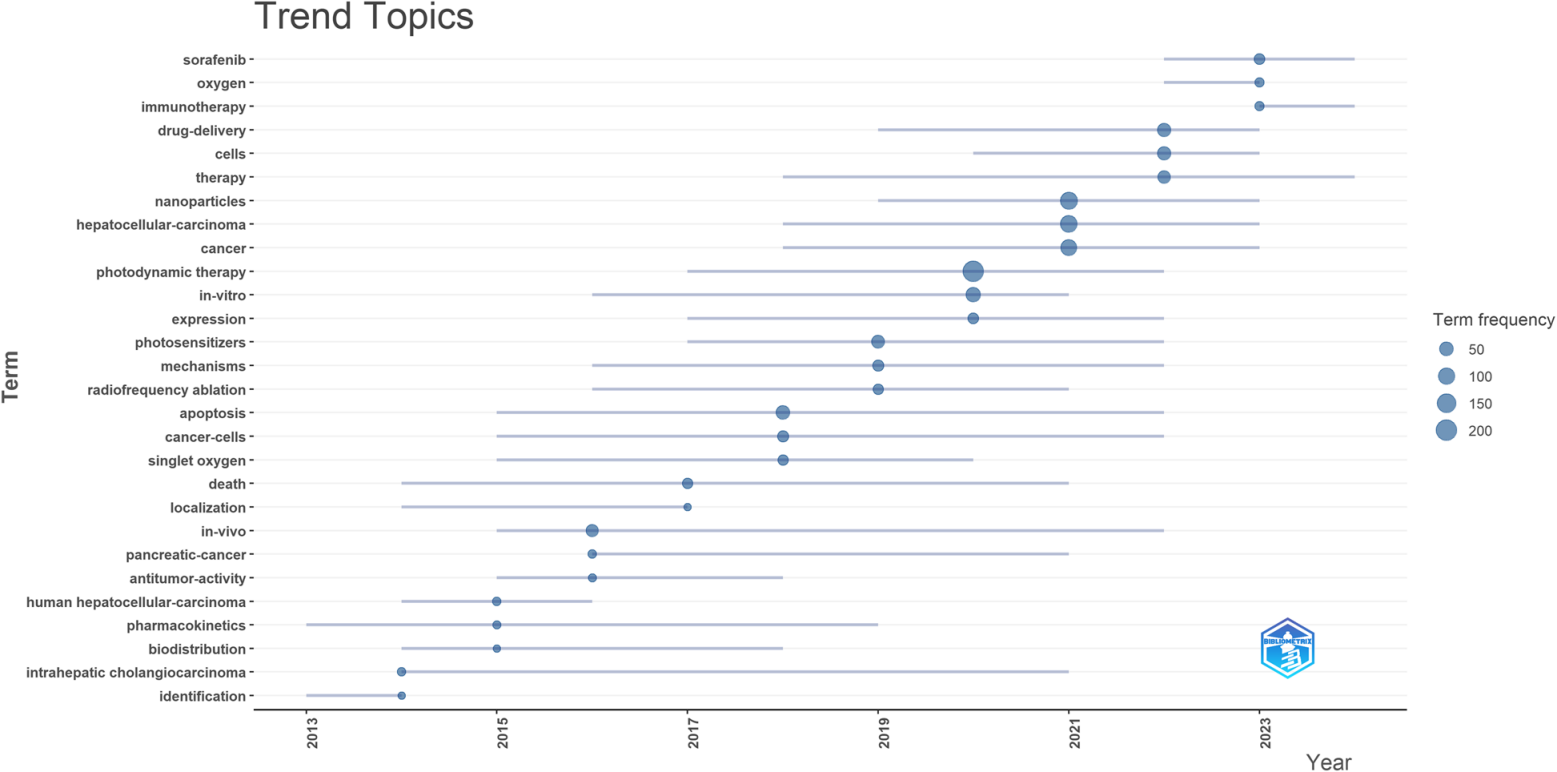
Trend topics on PDT for HCC

To analyze the developmental trends in this field from 2012 to 2025, we utilized the bibliometrix package within the R programming environment to construct a dynamic thematic evolution map (Fig. 7). The core methodology of thematic evolution mapping involves slicing and clustering the annual keyword co-occurrence network to summarize research trends for each period. Our findings indicate that the research on PDT for HCC has evolved through distinct phases, reflecting broader technological advances and clinical priorities: 2013–2015: Initial focus on molecular characterization of HCC and the in vivo metabolic behavior of PS. Research during this phase aimed to elucidate PDT mechanisms and optimize PS dosage through pharmacokinetic modeling to improve tumor targeting.2015–2021: Rapid adoption of nanocarriers, the emergence of multimodal systemic therapies, and deeper exploration of the multiple mechanisms underlying PDT-induced cell death.2021–2023: Significant advances in clinical translation and the development of individualized treatment plans, supported by robust in vivo and ex vivo experimental data.

The thematic evolution map suggests that the future of PDT for liver cancer will be closely tied to technological innovations such as nanotechnology. These tools are expected to drive further refinement in personalized PDT strategies, aligning with the broader movement toward precision medicine in oncology.

## Comprehensive analysis of hotspots

In conclusion, our comprehensive analysis—including citation burst detection, keyword frequency analysis, keyword clustering, and thematic evolution—has identified key emerging research hotspots at the intersection of PDT and HCC. Based on these findings, current research in this field is primarily concentrated in three areas: (1) elucidation of the mechanisms of PDT-induced apoptosis in HCC cells; (2) development of novel PS and the use of nanomaterials to enhance PDT efficacy; (3) synergistic integration of PDT with other therapeutic modalities. To facilitate the translation of PDT from experimental research to clinical application, future studies should place greater emphasis on exploring oxygen-independent therapeutic mechanisms, advancing multimodal nanoplatforms, and enhancing clinical translational potential.

## Discussion

### General information

We collected 547 relevant articles published between 2012 and 2025 from the WoSCC database. Overall, the number of publications in this field has shown a consistent upward trend. Among contributing countries, China produced the highest number of publications on PDT for HCC. In contrast, the United States exhibited the highest rate of international collaboration, likely reflecting its advanced scientific infrastructure and global appeal as a research partner. These 547 articles were published across 268 different academic journals. The International Journal of Nanomedicine and Photodiagnosis and PDT were the top two journals by publication volume. In terms of citations, Biomaterials ranked first by a significant margin, reaffirming its status as the leading outlet for disseminating influential research in this domain.

## Hotspots and development trends

### Mechanism of PDT-induced apoptosis in HCC cells

**Fig. 8:**
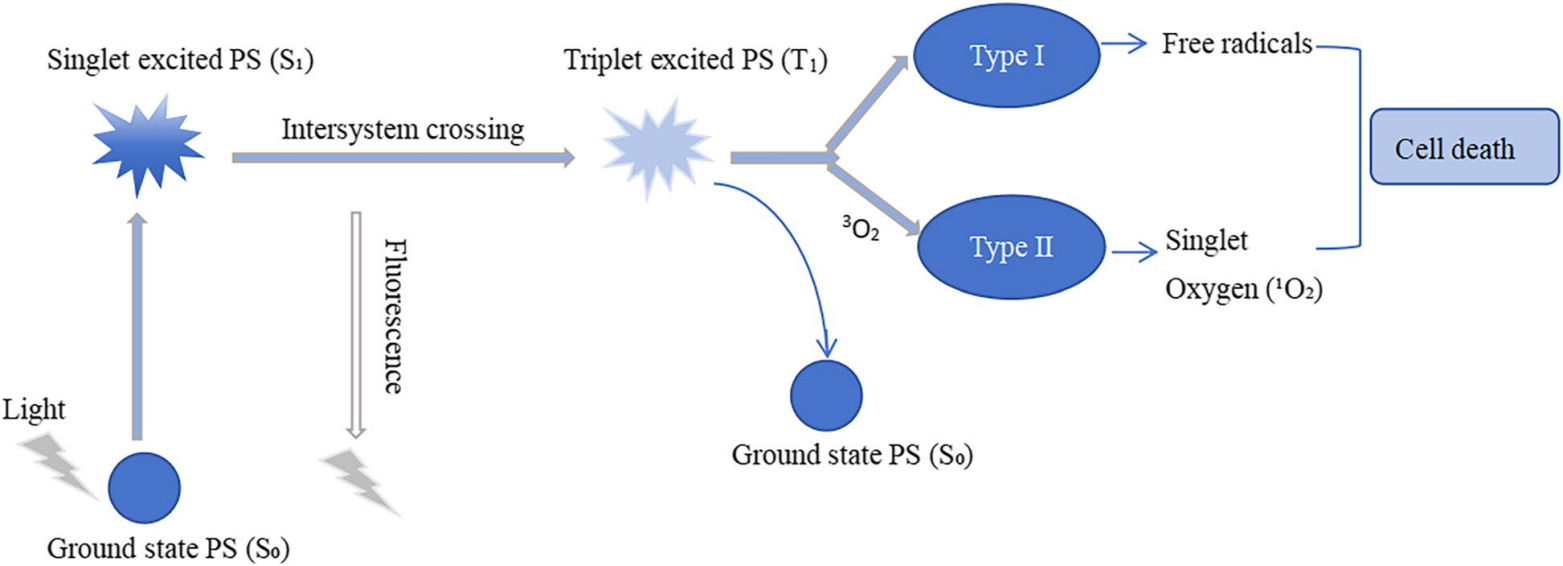
The basic principle of the photodynamic reaction

PDT is a targeted therapeutic technique based on a photochemical reaction and involves three main components: a PS, a light source of a specific wavelength, and tissue oxygen (O₂). Upon photon absorption, the PS transitions from its ground state PS (S₀) to the singlet excited PS (S₁). From S₁, it either returns to S₀ or undergoes intersystem crossing to the triplet excited PS (T₁). In the T₁ state, two photochemical pathways occur. Type I photochemistry: The T₁ undergoes a one-electron redox reaction with neighboring molecules, producing a free radical intermediate that subsequently generates ROS. Type II photochemistry: T₁ transfers energy to ground-state oxygen (^3^O₂), yielding singlet oxygen (^1^O₂), which can react with biomolecules—such as lipids, proteins, and nucleic acids—to induce oxidative damage. The basic principle of the photodynamic reaction is illustrated in Fig. 8.

PDT is believed to exert antitumor effects through three primary mechanisms: (1) Direct cytotoxicity toward HCC cells, where varying PDT intensities induce necrosis, apoptosis, or both [13]. (2) Destruction of tumor vasculature, wherein circulating PS in the vascular lumen causes endothelial damage via low-density lipoprotein (LDL) receptor-mediated endocytosis, leading to thrombosis and microvascular occlusion [14]. (3) Stimulation of antitumor immunity, whereby PDT-induced immunogenic cell death (ICD) releases damage-associated molecular patterns (DAMPs), triggering innate and subsequent adaptive immune responses [15].

Among these mechanisms, apoptosis is the most extensively studied in HCC. Apoptosis can be triggered via two main pathways: intrinsic and extrinsic. Activation of caspases, particularly caspase-3, −7, −8, and −9, is central to apoptotic signaling. ROS generated during PDT play a key role in initiating these cascades.

(1) Intrinsic Pathway.

The intrinsic apoptotic pathway is primarily activated by intracellular stress signals, such as ROS, and involves two key organelles: the mitochondria and the endoplasmic reticulum (ER).

Mitochondrial pathway: ROS damages the mitochondrial membrane, prompting the release of cytochrome c (Cyt-c) into the cytoplasm. Cyt-c then binds to Apaf-1 via its WD40 repeat domain, forming the apoptosome complex. The caspase activation and recruitment domain (CARD) of Apaf-1 interacts with the CARD of pro-caspase-9, leading to the activation of caspase-9. Subsequently, caspase-9 activates downstream effectors caspase-3 and caspase-7, initiating a caspase cascade and executing the classical apoptotic program [16, 17]. Additionally, oxidative stress induces upregulation of p53, which either activates pro-apoptotic factors within the mitochondria-mediated pathway or interacts with Bcl-2 family proteins to destabilize mitochondrial membrane potential [18, 19].

Endoplasmic reticulum stress (ERS) pathway: ROS disrupts ER homeostasis, leading to the activation of the unfolded protein response (UPR). When ER stress is excessive or prolonged and cellular homeostasis cannot be restored, UPR signaling initiates apoptosis by interfering with cellular metabolism and inducing mitochondrial dysfunction  [20]. ROS-mediated ER stress has also been shown to trigger immunogenic cell death in HCC, contributing to the elimination of residual tumor cells following chemotherapy [21].

(2) Extrinsic Pathway (Death Receptor Pathway).

Death receptors such as Fas, TRAIL-R1 (DR4), and TRAIL-R2 (DR5)—members of the tumor necrosis factor (TNF) receptor superfamily—play critical roles in extrinsic apoptosis [22]. TRAIL binds to DR4 or DR5, inducing receptor oligomerization and activation. This process recruits adaptor proteins such as Fas-associated death domain (FADD), which then bind to pro-caspase-8 to form the death-inducing signaling complex (DISC). Within this complex, pro-caspase-8 is activated to caspase-8, which initiates the apoptotic cascade [23]. One study showed that ROS can activate the JNK signaling pathway, upregulate death receptor expression, and enhance TRAIL-induced apoptosis in HCC cells  [24]. ROS also stimulates autophagy, further increasing DR expression on the tumor cell surface. Thus, elevating ROS levels in the tumor microenvironment (TME) may enhance tumor cell sensitivity to TRAIL, representing a promising strategy for combinational PDT-TRAIL therapy.

While apoptosis remains the dominant mode of PDT-induced cell death, emerging evidence also implicates additional forms of regulated cell death, such as necroptosis, ferroptosis, pyroptosis, parthanatos, and immunogenic cell death (ICD), in PDT’s antitumor activity [25, 26]. Further elucidation of these mechanisms may facilitate the clinical translation of PDT and its integration into multimodal therapeutic strategies for HCC.

## Development of new PS and application of nanotechnology to potentiate PDT

The number of studies investigating PDT for liver cancer remains comparatively limited relative to other tumor types, such as skin cancer. This discrepancy may be attributed to several factors, including the deep location of liver tissue within the body, which complicates access to effective PSs and suitable light sources. Additionally, liver tumors often exhibit sparse, disorganized vascularity and reduced oxygen supply. Nonetheless, PDT continues to garner considerable interest due to its high selectivity and specificity for tumor tissues, along with its low toxicity to surrounding normal tissues.

There are several possible reasons for the failure of PDT in the treatment of HCC. First, PS limitations: PS properties directly influence therapeutic outcomes. For example, ZnPc can exhibit concentration-dependent cytotoxicity at high doses [27]. and complex formulations—such as AlPcS₄ encapsulated in gold nanorods—pose translational challenges due to elaborate synthesis procedures [28]. Second, TME barriers: hypoxia within HCC tumors reduces ^1^O₂ generation during PDT, thereby diminishing its effectiveness [29]. Moreover, elevated GSH levels in the TME scavenge ROS^[30]^, further attenuating oxidative damage. Third, technical and translational hurdles: the long-term biosafety of nano-PS constructs such as PTPEDC2 remains unestablished, hindering clinical adoption  [31].

To address these limitations, efforts are focused on developing novel photosensitizers, enhancing light penetration, and designing efficient delivery systems for PSs. PSs can sequentially direct HCC cells to initiate programmed death cascades through precise actions at the molecular level (e.g., modulation of cell membrane permeability behavior, mediation of reactive oxygen species signaling). Over several decades of research, PSs have evolved into three generations: First-generation PSs, including hematoporphyrin (Hp) and its derivatives (HpD), laid the foundation for PDT. Porfimer sodium was the first PDT drug approved for clinical use [32, 33]. Second-generation PSs are synthetic compounds derived from or related to porphyrins, bacteriochlorins, phthalocyanines, chlorins, benzoporphyrins, curcumin, and methylene blue derivatives. These agents offer improved photophysical characteristics, such as reduced phototoxicity under visible light, faster metabolic clearance, and shorter intervals between administration and light exposure. However, they still present limitations, including poor blood stability, rapid systemic clearance, poor water solubility, low bioavailability, limited tumor selectivity, and a high dependency on oxygen. Third-generation PSs involve chemical conjugation with tumor-targeting moieties (e.g., proteins, peptides, glycans, or antibodies) or encapsulation in delivery systems such as liposomes, micelles, or nanoparticles. These strategies enhance the selective accumulation of PSs at tumor sites while addressing the shortcomings of second-generation agents, such as inadequate solubility, lack of specificity, and poor intracellular transport [34, 35].

An ideal PS for HCC treatment should possess strong tumor selectivity and efficient absorption within the therapeutic window for deeper tissue penetration, while retaining sufficient photonic energy to activate itself and induce a vigorous photodynamic response [36]. Table 7 summarizes the top 25 citations by burst strength and the 20 most-cited articles on the types of PSs used for the treatment of HCC, preparation methods, therapeutic mechanisms, and advantages and disadvantages.

**Table 7:** Photosensitizer types, preparation methods, therapeutic mechanisms, and advantages and disadvantages

PS	Type	Preparation method	Therapeutic mechanism	Therapeutic Advantages	Limitations	Liver cancer model	References
Indocyanine Green (ICG)	Near-infrared (NIR) fluorescent dye	Direct administration	1. Thermal effects lead to protein denaturation and cell membrane disruption; 2. ROS activate apoptotic pathways through oxidative damage to mitochondria and DNA	1. High tumor selectivity: ICG specifically accumulates in HCC cells via organic anion-transporting polypeptide (OATP), with a tumor-to-background fluorescence intensity ratio as high as 255:1, which significantly reduces damage to normal tissues. 2. Dual therapeutic effects of PTT and PDT3. Repeated irradiation enhances anti-tumor efficacy. 3. Repeated irradiation enhances anti-tumor efficacy. 4. High safety profile with very low incidence of adverse reactions (< 0.01%)	1. Depth of penetration limitation: NIR light penetrates to a depth of about 10 mm and is only indicated for superficial tumors (e.g., liver surface or peritoneal metastases). 2. Wavelength not optimized: The NIR laser wavelength used in the experiment was 823 nm, while the peak absorption of ICG in vivo is 805 nm, and the incomplete match may affect the efficacy	Huh-7 cell nude mouse transplantation model (in vivo animal experiments, not clinical experiments)	[ ^36^ ]
Al(III) phthalocyanine chloride tetrasulfonic acid (AlPcS4)	Phthalocyanine-based	Encapsulated in mesoporous silica-coated gold nanorods (MSGNR), with β-cyclodextrin gatekeepers via Pt (IV)	AlPcS₄ photoexcitation generates ROS, GNR photothermal ablates tumors and promotes drug penetration, and platinum (IV) reduction to cisplatin inhibits DNA repair	1. Combines PDT, PTT, and platinum-based chemotherapy. 2. LA ligand specifically binds to galactose receptors overexpressed on the surface of HCC cells, increasing tumor accumulation. 3. High concentrations of GSH in the tumor trigger the reduction of Pt(IV) to cisplatin and the release of AlPcS₄, synergizing the effects of chemotherapy with PDT. 4. The Photothermal effect of GNR enhances PDT efficacy	1. Complex preparation of gold nanorods. 2. Dual laser requirements. 3. Potential long-term toxicity and removal issues	HepG2 xenograft model in nude mice; HepG2 cell line (In vivo animal experiments and in vitro cellular experiments, non-clinical)	[ ^28^ ]
Rose Bengal (RB)	Organic dye-based PS	Co-loaded with Erastin into CD47-overexpressing exosomes via sonicatio; final formulation: Er/RB@ExosCD47	The dual mechanism of ROS generated by RB laser excitation and Erastin iron death synergizes against tumors	1. RB laser excitation generates ROS, combined with Erastin iron death synergistically enhances anti-tumor effects. 2. CD47-modified exosomes escape immune clearance and enhance tumor accumulation. 3. low toxicity	1. complex and costly to prepare. 2. Unproven long-term safety. 3. Still reliant on laser exposure	Hepa1-6-luc cells of the HCC xenograft model; Hepa1-6 mouse HCC cell line (In vivo animal experiments and in vitro cellular experiments, non-clinical)	[ ^40^ ]
Chlorin e6 (Ce6)	Chlorin derivative	Loaded into light-activatable liposomes with Pt (IV); GSH-responsive drug release	Ce6 produces ROS upon light irradiation, and glutathione (GSH) depletion enhances ROS	1. Combines PDT with chemotherapy. 2. Improves immunomodulation. 3. reduces tumor hypoxia	1. Dependency on repeated light exposure. 2. High liposome stability is required	Patient-derived HCC xenograft model (Human tumor xenograft (PDX) trial, non-clinical)	[ ^29^ ]
PTPEDC2	Conjugated polymer aggregation-induced emission (AIE) PS	Encapsulation of PTPEDC2 into nanoparticles with DSPE-PEG-Mal (AIE PS dots)	Dual-photon excitation efficiently generates active oxygen to precisely kill deep-seated tumors	1. Highly effective two-photon photodynamic therapy (2PE-PDT). 2. High ^1^O_2_ yield and high 2PA cross-Sect. 3. Enhanced intratumoral accumulation and targeted delivery due to TAT-modification 4. Enables deep tissue imaging and spatially confined therapy	1. complex and costly to prepare. 2. Unproven long-term biosafety	In vitro model: HeLa cells; In vivo model: zebrafish liver tumor model (In vivo animal experiments and in vitro cellular experiments, non-clinical)	[ ^31^ ]
TPE-red-2AP2H	Red-emissive AIE luminogen based on tetraphenylethylene (TPE)	TPE-red-2AP2H was made by blending AP2H (IHGHHIISVG) into TPE-red	Targeting LAPTM4 B turns on fluorescence and light activation, generating ^1^O_2_ to induce apoptosis	1. Dual function: cancer cell-specific fluorescence imaging and PDT 2. Activatable red fluorescence reduces background noise 3. Strong photostability 4. High selectivity for tumor cells via LAPTM4 B targeting, minimal phototoxicity to normal cells	1. Insufficient in vivo validation. 2. Long-term safety not defined. 3. May be limited by LAPTM4 B expression variability across tumor types	HepG2 liver cancer cell model (in vitro cellular experiments, non-clinical)	[ ^41^ ]
Zinc (II)-phthalocyanine (ZnPc)	Second-generation phthalocyanine derivative	Upconversion nanoparticles covalently bound to zinc phthalocyanine (UCNPs-ZnPc/FA)	UCNPs induce apoptosis and necrosis in tumor cells by excitation of ZnPc to produce ^1^O_2_ via fluorescence resonance energy transfer (FRET)	Near-infrared excitation: large 980 nm light penetration depth reduces tissue damage. 2. Targeting: folic acid (FA) modification enhances tumor targeting for imaging-guided PDT. 3. 80.1% tumor growth inhibition at only 351 J/cm^2^ (0.39 W/cm^2^). 4. Significantly increased ^1^O_2_ yield	1. slight cytotoxicity was present at high concentrations (> 200 μg/mL). 2. The long-term biocompatibility and in vivo metabolic pathways of the nanoparticles were not fully verified	Hepa1-6 cell subcutaneous graft tumor model (in vitro cellular experiments, non-clinical)	[ ^27^ ]
TPETS (Tetraphenylethene derivative with Aggregation-Induced Emission)	AIE-type organic PS	TPETS encapsulated in DSPE-PEG-Mal via nano-precipitation. Conjugated with cRGD (cyclic peptide) via thiol–maleimide click reaction to produce T-TPETS nanodots	1. AIE properties enhance ROS generation. 2. Induces cell death via the mitochondrial apoptotic pathway. 3. Red light imaging to guide precise treatment in real time	High brightness, efficient ROS generation. 2.targeted imaging. 3. Targeted enhancement of tumor-specific accumulation	1. complex preparation. 2. unknown long-term metabolism. 3. challenging clinical translation	In vitro model: HepG2 liver cancer cell;In vivo model: HepG2 cell subcutaneous xenograft nude mouse model (In vivo animal experiments and in vitro cellular experiments, non-clinical)	[ ^42^ ]

In addition to innovations in PS, nanoscale drug delivery vehicles—such as nanoparticle systems and liposomes—and electroporation (EP) are emerging as important technological tools to advance this therapy [37]. Nanoparticles offer significant advantages in protecting PS from enzymatic degradation, enabling targeted delivery, and facilitating controlled release [38]. Delivery systems—including liposomes, gold nanoparticles, and upconversion nanoparticles—have been employed in nonclinical in vivo and in vitro studies of liver cancer (Table 7). EP, as a physical modality, can enhance the therapeutic efficacy of PDT by increasing cell membrane permeability, opening microvasculature, and alleviating tumor hypoxia. Abd et al. [39]. developed a microneedle-based delivery platform combining ion introduction (MN-IP) and EP technologies with PDT, sonodynamic therapy (SDT), and sonophotodynamic therapy (SPDT) to form an MN@IP@EP@TDDG system; they used a macroalgae-derived sensitizer to treat EAC-bearing mice, achieving a favorable therapeutic effect.

From the data presented, it is evident that the integration of nanotechnology into PDT offers several notable advantages for HCC management: (1) enhanced efficacy of PSs; (2) improved tumor tissue penetration; (3) targeted drug delivery at the tissue, cellular, or subcellular level—as exemplified by platforms such as CD47-functionalized exosomes (Er/RB@Exos^CD47)^[29]^, which represent an innovative approach to targeted delivery; (4) enabling sustained drug release; (5) tumor-targeted imaging capabilities. Collectively, these innovations serve to optimize PDT and support the advancement of precision medicine in liver cancer treatment.

## Synergistic treatment of PDT with other therapies

PDT is particularly valuable as a complementary treatment for early-stage HCC in patients who are unsuitable for surgery or ablation, for lesions located near major blood vessels or bile ducts where local treatments pose higher risks, and for managing residual or recurrent lesions following surgery. PDT has the following potential advantages for unresectable HCC:(1) Minimally invasive treatment of superficial lesions: ICG is suitable for hepatic surface metastases and peritoneal nodules, as it has high tumor selectivity and dual PTT/PDT effects [36]. (2)Combination therapy: Fan et al. [43]. developed IR820-GPC3-Gd NPs (IGD NPs), which can be GPC3-targeted and enriched in tumor tissue, and can synergistically treat mid-advanced HCC with PTT and PDT, and precisely delineate tumor boundaries via highly sensitive fluorescence/MRI. (3) Targeting deeper tissues: Conjugating ZnPc with upconversion nanoparticles (UCNP-ZnPc/FA) allows 980 nm NIR excitation to penetrate deeper tissues. Two-photon excitation of PS (PTPEDC2) enables precise deep-tissue PDT, but clinical validation is still needed^[31]^. Clinically, PDT is often integrated into multimodal treatment regimens to enhance therapeutic efficacy against HCC. Next, we focus on the synergistic treatment of PDT with other therapies.

The following studies are in preclinical stages: Yavaş A et al. [44]. found that sonodynamic therapy (SPDT) with nano-TiO₂/Pc induced oxidative stress and activated apoptosis pathways, raising HepG2 cell apoptosis to 83.80%. It showed more cell toxicity than single therapy. But this study is only in the in vitro cell research stage, and its in vivo therapeutic effects need further validation. Li et al. [45]. developed an ICG&Cur@MoS₂ nanoplatform, where ICG and curcumin-loaded layered MoS₂hollow spheres delivered synergistic photothermal—photodynamic therapy against HCC. In vitro and in vivo studies showed it shrank tumors significantly with relatively low systemic toxicity. However, its clinical translation potential requires further exploration. Yu et al. [46]. developed ZnPc/SFB@BSA, BSA-coated ZnPc and sorafenib nanocapsules. In—vitro and in—vivo experiments showed it worked well in PDT—PTT—chemotherapy against HCC. Under near-infrared light, ZnPc/SFB@BSA can suppress HCC cell proliferation and metastasis, and induce apoptosis. It not only remarkably reduces tumor volume and weight in vivo, but also shows good biocompatibility and low toxicity. Still, this study is in the in vitro and in vivo stage. Wang et al.^[47]^ Created a multifunctional nano-platform combining PDT, PTT, CDT, and GAT for HCC therapy. Radiotherapy (RT) uses high-energy ionizing radiation to induce DNA damage, thereby triggering apoptosis and necrosis. A key advantage of RT is its ability to treat tumors regardless of tissue depth. However, its limitations include potential damage to adjacent healthy tissues and reduced efficacy in hypoxic TMEs. Furthermore, the antioxidant defense systems within cancer cells can diminish the cytotoxic effects of ionizing radiation. Combining RT with PDT has been shown to enhance therapeutic outcomes by increasing ROS production, leading to greater tumor cell destruction and improved radiation efficacy [18].

Many researchers have also explored clinical applications of PDT for HCC. Cheng et al. [49]Conducted a retrospective clinical study on 60 HCC patients and found that conventional chemotherapy combined with HPD-based nanomedicine PDT improved treatment efficacy and reduced liver injury. In a multicenter phase I clinical trial [50]. assessing mTHPBC—mediated PDT for irresectable colorectal liver metastases, 24 patients received intravenous mTHPBC at 0.6 or 0.3 mg/kg, then 740—nm laser irradiation 48 or 120 h later, and all lesions necrosed within one month; the laser treatment caused mild pain and transient subclinical liver toxicity, with phlebitis and skin phototoxicity in the 0.6 mg/kg group. In a prospective clinical study [51]. of 11 patients with bile duct—invaded unresectable HCC, after intravenous injection of 2 mg/kg photosensitizer (Photogem or Photofrin), PDT was performed 48 h later with intracavitary 630—nm light irradiation (180 J/cm) and bile duct drainage, leading to shrinkage or disappearance of intrahepatic bile duct cancer lesions and improved biliary patency in some patients. All of these studies have confirmed the safety and efficacy of PDT in the treatment of HCC.

## Strengths and limitations

Despite employing a rigorous and well-established methodology to explore the application of PDT in HCC, this study has several limitations that should be acknowledged: (1) Database selection: This study relied solely on data from the Web of Science Core Collection (WoSCC). Although WoSCC is widely recognized as one of the most comprehensive and authoritative databases for bibliometric analysis^[52]^, exclusive use of this source may have resulted in the omission of relevant literature indexed in other databases. (2) Search strategy: The search was limited to English-language publications, which may introduce language bias and limit the generalizability of the findings, especially considering the global distribution of PDT research. (3) Author analysis: Author-level bibliometric analysis was not performed, primarily due to potential inaccuracies arising from name duplication, particularly common among authors from China. To avoid erroneous attribution and misinterpretation, this component was excluded from the current study. (4) Tool-based analysis limitations: The analytical tools used—CiteSpace and VOSviewer—offer powerful visualization and trend analysis capabilities, but they cannot replace systematic reviews or critical appraisal of individual studies. Bibliometric indicators such as citation frequency are inherently time-sensitive; newer publications may receive fewer citations simply due to their recent publication date, rather than lower relevance or quality^[53]^.

While these limitations are largely inherent to the nature of bibliometric research, the conclusions drawn from this study remain robust and offer valuable insights for future academic investigations in the field of PDT and HCC.

## Conclusion

In this study, we used bibliometrics to analyze major research hotspots and trends in PDT for HCC. Current hotspots include: (1) mechanisms of PDT-induced cell death in HCC; (2) development of novel photosensitizers; (3) application of nanomaterials to enhance PDT efficacy; (4) synergistic combinations of PDT with other therapeutic modalities. These focus on addressing PDT's limitations, such as inadequate tumor targeting, limited tissue penetration, and tumor hypoxia, to advance precise medical targeting drug delivery systems. In summary, this study provides a comprehensive overview of the current research landscape and emerging focal areas in the application of PDT for HCC. The findings enhance our understanding of prevailing research directions and offer a foundation for guiding future investigations. Notably, the integration of nanotechnology into PDT has emerged as a key area of interest due to its potential for precise targeting, multimodal diagnostic and therapeutic capabilities, and the ability to overcome drug resistance. However, challenges remain, particularly in terms of the high production costs of nanomaterials and the complexities associated with clinical translation on a large scale.

## Supplementary Information

Supplementary Material 1.

Supplementary Material 2.

Supplementary Material 3.

## Data Availability

No datasets were generated or analysed during the current study.
